# Impact of hepatitis B vaccination on acute hepatitis B epidemiology in European Union/European Economic Area countries, 2006 to 2014

**DOI:** 10.2807/1560-7917.ES.2018.23.6.17-00278

**Published:** 2018-02-08

**Authors:** Alessandro Miglietta, Chantal Quinten, Pier Luigi Lopalco, Erika Duffell

**Affiliations:** 1 European Centre for Disease Prevention and Control (ECDC), Stockholm, Sweden; 2 Central Tuscany Health Authority, Units of Epidemiology and Preventive Medicine & Epidemiologic Observatory of the Regional Health Agency of Tuscany, Florence, Italy; 3 Department of Translational Research on New Technologies in Medicine and Surgery, University of Pisa, Italy

**Keywords:** Hepatitis B, Surveillance, public health policy, Epidemiology, vaccination

## Abstract

Hepatitis B prevention in European Union/European Economic Area (EU/EEA) countries relies on vaccination programmes. We describe the epidemiology of acute hepatitis B virus (HBV) at country and EU/EEA level during 2006–2014. Using a multi-level mixed-effects Poisson regression model we assessed differences in the acute HBV infection notification rates between groups of countries that started universal HBV vaccination before/in vs after 1995; implemented or not a catch-up strategy; reached a vaccine coverage ≥ 95% vs < 95% and had a hepatitis B surface antigen prevalence ≥ 1% vs < 1%. Joinpoint regression analysis was used to assess trends by groups of countries, and additional Poisson regression models to evaluate the association between three-dose HBV vaccine coverage and acute HBV infection notification rates at country and EU/EEA level. The EU/EEA acute HBV infection notification rate decreased from 1.6 per 100,000 population in 2006 to 0.7 in 2014. No differences (p > 0.05) were found in the acute HBV infection notification rates between groups of countries, while as vaccine coverage increased, such rates decreased (p < 0.01). Countries with universal HBV vaccination before 1995, a catch-up strategy, and a vaccine coverage ≥ 95% had significant decreasing trends (p < 0.01). Ending HBV transmission in Europe by 2030 will require high vaccine coverage delivered through universal programmes, supported, where appropriate, by catch-up vaccination campaigns.

## Introduction

Hepatitis B is caused by the hepatitis B virus (HBV), an enveloped DNA virus that infects the liver and causes cirrhosis and hepatocellular carcinoma [1]. Primary HBV infection in susceptible individuals can be either symptomatic or asymptomatic, the latter being often the case. Hepatitis B surface antigen (HBsAg) is the earliest marker of hepatitis B infection, and is widely used in seroprevalence surveys to estimate the number of infected people and as indicator of transmission risk [2].

Although most primary acute infections are self-limiting, their case fatality rate is 0.5–1%. The risk of developing chronic HBV infection is dependent on age of infection,******and for infants infected during the first year of life, it is estimated at 80–90% [1].

HBV is widely prevalent and it is estimated that approximately one third of the world’s population has been exposed to the virus, with 250 million people chronically infected [2].

Global coverage with three doses of hepatitis B vaccine in children increased from 30% in 2000 to 82% in 2014 [3], but a large proportion of the global population has not been reached through vaccination programmes and remains unprotected with a risk that many of those who will become infected will in turn become potentially infectious. Every year, more than 780,000 people die worldwide due to complications of hepatitis B, mostly from cirrhosis and liver cancer [4]. The World Health Organization (WHO) European Region is considered to be an intermediate endemicity region with an HBsAg prevalence of 1.6% ranging from < 0.1% in the United Kingdom (UK) to 10.3% in Kyrgyzstan [2].

In 2011, the European Centre for Disease Prevention and Control (ECDC) introduced an enhanced surveillance programme for hepatitis B across European Union/European Economic Area (EU/EEA) countries [5], using the European Surveillance System (TESSy), a platform for submission, warehousing and retrieval of web-based data on communicable diseases under EU surveillance. Since then, the collection of data on acute and chronic infections has taken place on an annual basis and the database includes reported cases dating back to 2006. Data analysis has highlighted differences in the reported numbers and rates of acute HBV infections between countries [6]. The data also suggest a downward trend in the acute HBV infections during 2006–2014 in many countries, which is most likely due to the impact of widespread implementation of vaccination programmes.

The main prevention strategy for hepatitis B is vaccination, through universal, targeted and catch-up programmes [7]. In 1992, the World Health Assembly recommended the inclusion of HBV vaccine in all national immunisation programmes [8]. In 2016 the Assembly approved the Global Health Sector Strategy on viral hepatitis, 2016–2021, which sets the goal of eliminating viral hepatitis as a major public health threat by 2030, by using the WHO guidance: inclusion of hepatitis B virus vaccine in national childhood immunisation schedules; implementation of catch-up hepatitis B virus vaccination strategies; and achievement of 90% vaccine coverage (third dose coverage) [9]. To date, most of the European countries have implemented a universal HBV vaccination programme, but national differences exist in the type of strategy used and the vaccine coverage achieved [10].

Studies conducted in Europe, at national and local level, have indicated that HBV vaccination programmes have resulted in the reduction of acute hepatitis B incidence [11-14]. However, there is no recent information on the impact of the different implemented HBV vaccination strategies and the vaccine coverage achieved, on the epidemiological trends of acute HBV at national and EU/EEA level, with analysis of the differences between countries.

The aim of this study is to describe the epidemiological trend of the acute HBV infection notification rate at country and EU/EEA level during 2006–2014 and to assess the possible impact of the different HBV vaccination strategies implemented by the EU/EEA countries on these rates. We also evaluated the impact of the vaccine coverage levels and estimates of HBsAg prevalence in the general population on the acute HBV infection notification rate over this period, to further guide countries’ actions to achieve the goal of eliminating viral hepatitis by 2030.

## Methods

### Data sources

Acute HBV infection notification data submitted to ECDC from EU/EEA countries were obtained for the time period 2006–2014 from TESSy. Country population denominator data (2006–2014) were obtained from Eurostat [15]. For the UK, population data from the Office for National Statistics were used to exclude Scotland from the denominator as no hepatitis B data from Scotland were ever reported to ECDC. Over the time period 2005–2013, data on HBV vaccine coverage for the three doses (HepB3 coverage) were obtained from WHO’s Centralized Information System for Infectious Diseases (CISID) [16].

### Definitions

A number of factors were hypothesised to potentially influence the acute HBV infection notification rates. These included the time when a HBV universal vaccine programme (e.g. childhood vaccination programme) was introduced, the vaccination coverage level achieved from this programme, the presence of any accompanying catch-up vaccination strategies (e.g. additional vaccination campaigns targeting individuals in a defined age group, with the objective of reaching a high proportion of susceptible individuals) and the background HBsAg prevalence. To assess the impact of these different factors on the acute HBV infection notification rates, EU/EEA countries were grouped according to four binary criteria (Table 1). 

**Table 1:** Grouping of European Union/European Economic Area countries according to four binary criteria, 2006–2014 (n = 17 countries^a^)

Criterion	Description	Countries
1	Started hepatitis B universal vaccination programme before/in 1995 (coded as 1)	Bulgaria, France, Germany, Romania
	Started hepatitis B universal vaccination programme after 1995 (coded as 0)	Austria, Czech Republic, Estonia, Greece, Hungary, Ireland, Latvia, Lithuania, Malta, the Netherlands, Poland, Slovakia, Slovenia
2	Had any kind of catch-up hepatitis B vaccination programme (coded as 1)	Bulgaria, Estonia, France, Germany, Latvia, Lithuania, Malta, Poland, Romania, Slovakia
	Had no catch-up hepatitis B vaccination programme (coded as 0)	Austria, Czech Republic, Greece, Hungary, Ireland, the Netherlands, Slovenia
3	Had three doses hepatitis B vaccine coverage ≥ 95% (coded as 1)	Bulgaria, Czech Republic, Greece, Ireland, Latvia, the Netherlands, Poland, Romania, Slovakia
	Had three doses hepatitis B vaccination coverage < 95% (coded as 0)	Austria, Estonia, France, Germany, Lithuania, Malta, Slovenia
4	Had HBsAg prevalence among general population ≥ 1% (coded as 1)	Austria, Bulgaria, Greece, Hungary, Latvia, Lithuania, Poland, Romania
	Had HBsAg prevalence among general population < 1% (coded as 0)	Czech Republic, Estonia, France, Germany, Ireland, the Netherlands, Slovakia, Slovenia

HBsAg: Hepatitis B surface antigen.

^a ^27 European Union/European Economic Area countries were included in the study. Of these 27 countries, Cyprus, Luxembourg, Portugal and Spain (n = 4) were excluded in the analysis presented in the Table because they did not report consistently hepatitis B virus infection notification data for at least 5 years, and Denmark, Finland, Iceland, Norway, Sweden and the United Kingdom (n = 6) were excluded because they did not have a universal vaccination programme between 2006 and 2014. In addition, Hungary was excluded from criteria 3 because this country did not report vaccination coverage data on Centralized Information System for Infectious Diseases (CISID), and Malta was excluded from criteria 4 because HBsAg prevalence data were not available for Malta in the systematic reviews considered for this study [2,19].

We used the review of Lernout et al. [10] and the results from the Vaccine European New Integrated Collaboration Effort (VENICE) II project [17] to group the countries according to the criterion number 1 (countries that started universal HBV vaccination programme before/in vs after 1995; the median year of HBV vaccine introduction among the analysed countries) and criterion 2 (countries with vs without a catch-up HBV vaccination programme). In order to group the countries according to criterion number 3 (countries with HepB3 coverage ≥ 95% vs  < 95%; target of the WHO European Action Plan for viral hepatitis [18]), the information from CISID on HepB3 coverage (measured at 1 year of age) was used including the last available data from each country. Finally, to group the countries according to criterion number 4 (countries with HBsAg prevalence among general population < 1% vs ≥ 1%; target of the Global Health Sector Strategy on viral hepatitis, 2016–2021 [9]), we used the systematic reviews of Kowdley et al. [19] and Schweitzer et al. [2].

### Descriptive analysis

To describe the epidemiological trend of acute HBV infection between 2006 and 2014, notification rates per 100,000 inhabitants were calculated at both the EU/EEA and national level. At EU/EEA level, the annual acute HBV infection notification rate was calculated, including only population denominator data from countries that provided data on acute HBV for that year. For this analysis all countries that reported data on acute HBV were included.

We also described the epidemiological trend of acute HBV infection by group of countries assigned to each of the four grouping criteria. For each of these, notification rates per 100,000 inhabitants were calculated using the sum of the annual acute HBV cases reported by each group of countries as the numerator and the sum of the countries’ group population by year as the denominator. For this analysis countries without a universal HBV vaccination programme between 2006 and 2014 were excluded (Denmark, Finland, Iceland, Norway, Sweden and UK). In addition, Hungary was excluded from criteria 3 because this country did not report vaccination coverage data on CISID, and Malta was excluded from criteria 4 because HBsAg prevalence data were not available in the systematic reviews [2,19]. Finally, using the ECDC Map Maker tool (EMMA) [20] we presented the average acute HBV infection notification rate over the period 2006–2014 by reporting country.

### Statistical analysis

#### Differences in HBV infection notification rates between country groups by criterion

We used a multi-level mixed-effects Poisson regression model to evaluate statistically significant differences between two group of countries in their acute HBV infection notification rates (trends) by each criterion/strategy separately (univariate analysis) and combined to account for possible confounding between criterion/strategies (multivariate analysis) [21,22]. Time was modelled as change over time (trend), while countries were included as clusters/random effect. In this analysis country-data were combined together in order to create two groups of countries (within a criterion) to compare. The output is whether there was a statistically significant difference in the acute HBV infection trend (during 2006–2014) between two groups of countries: one that implemented a strategy and one that did not.

For this analysis the countries without universal HBV vaccination programme were excluded as well as countries that did not report consistently acute HBV data for at least 5 years (Cyprus, Luxembourg, Portugal, Spain); in addition, Hungary and Malta were excluded from the groups based on criteria 3 and 4, respectively. The analysis was carried out using the number of acute HBV infections as the dependent variable, the country population as the exposure variable and the strategies as the independent variables.

#### Correlation between HepB3 coverage and acute HBV infection notification rate at country- and EU/EEA-level 

In addition, we assessed the association/correlation between HepB3 coverage and acute HBV infection notification rates for each country separately and at EU/EEA level during 2006–2014, using Poisson regression models. At country level, the number of reported acute HBV infections in each year, was used as the outcome variable, the national HepB3 coverage (coded as a percentage) for the previous year as predictor variable and the country population being the exposure variable. As in previous studies [23,24], we hypothesised that the effects of vaccination programmes may be delayed until the following year. At the EU/EEA level, the annual HepB3 coverage was calculated as the mean of included countries; we excluded those countries that did not report both acute HBV cases and HepB3 coverage data for at least five years (Cyprus, Denmark, Finland, Hungary, Iceland, Ireland, Luxembourg, the Netherlands, Norway, Portugal, Spain, Sweden and UK).

Differently from the multi-level mixed-effects Poisson analysis, here each country is evaluated separately to assess the correlation between the yearly country-acute-HBV infection notification rate and the related country-vaccine coverage (inputted as percentage and not as 0 = < 95% vs 1 = ≥ 95% as in the multilevel-mixed effects Poisson regression model). The model gives the output taking in consideration the whole period under study, and is interpreted as the reduction in the acute HBV rates for each percentage point increase in the vaccine coverage. So, differently from the multi-level mixed-effects regression model, there is not a comparison between two groups/trends, but the evaluation of any statistical correlation between a rate and a percentage at country level, without interactions with data of other countries.

For both the Poisson regression analyses, results were expressed in terms of incidence rates ratio (IRR) with 95% confidence interval (CI). The significance level was set at p < 0.05. STATA 13 was used for the data analysis.

#### Detecting trend changes in acute HBV infection notification rates in groups of countries by defined criteria

Finally, we used Joinpoint (JP) regression models in order to identify statistically significant trends and changes in trends (increasing/decreasing) in the acute HBV infection notification rate during 2006–2014 by group of countries assigned to each of the four grouping criteria. This method describes changes in data trends by connecting several different line segments on a log scale at ‘JPs’, and can identify points where a statistically significant change over time in the linear slope of the trend occurred [25]. The analysis starts with the minimum number of JPs (i.e. 0 JP, which is a straight line) and tests whether one or more JPs are statistically significant and must be added to the model. The tests of significance use a Monte Carlo permutation method. In addition, an annual percentage change (APC) for each line segment is estimated. The APC is tested to determine if it is different from the null hypothesis that the APC is 0%. In the final model, each JP indicates a statistically significant change in trends (increase or decrease) and each of those trends is described by an APC. The average annual percentage change (AAPC) and the related 95% CI are also computed in order to have a summary measure of the trend over the complete period of study. AAPC indicates whether the trend showed a statistically significant increase or decrease during a fixed predetermined interval. The AAPC is a weighted average of the APCs, with the weights equal to the length of the JP segments.

In our study the APC is interpreted as the differences (increase or decrease) in the acute HBV infection notification rate between JP segments when a JP is found, while the AAPC indicates whether the acute HBV infection notification rate trend significantly increased or decreased during the fixed predetermined interval 2006–2014 [25]. For this analysis the six countries without universal HBV vaccination programme were excluded, and for criteria 3 and 4, respectively, Hungary and Malta were excluded.

JP Regression Programme version 4.0.4 was used to carry out the analysis.

## Results

Of 31 EU/EEA countries, Belgium, Croatia and Italy were excluded from this study because data reported could not distinguish between acute or chronic hepatitis. Moreover no data were available from Liechtenstein. Among the 27 remaining countries, which were all part of the study, a total of 32,949 acute HBV cases were reported during the period 2006–2014. While the UK contributed data, Scotland was excluded from the UK because this country was unable to provide any data.

The number of countries reporting data to ECDC increased from 19 in 2006 to 27 in 2014. Most of the 27 countries reporting data in 2014 (n = 21) have implemented a universal HBV vaccination programme, with 16 introducing this programme after 1995. Among the 21 countries with a universal programme, 12 also adopted catch-up immunisation strategies. Eleven countries have an estimated HBsAg prevalence among general population ≥ 1%; six of these eleven countries are located in the eastern part of Europe (Bulgaria, Hungary, Latvia, Lithuania, Poland, Romania).

### Description of notification rates

#### Notification rates at country and EU/EEA level

**Figure 1:**
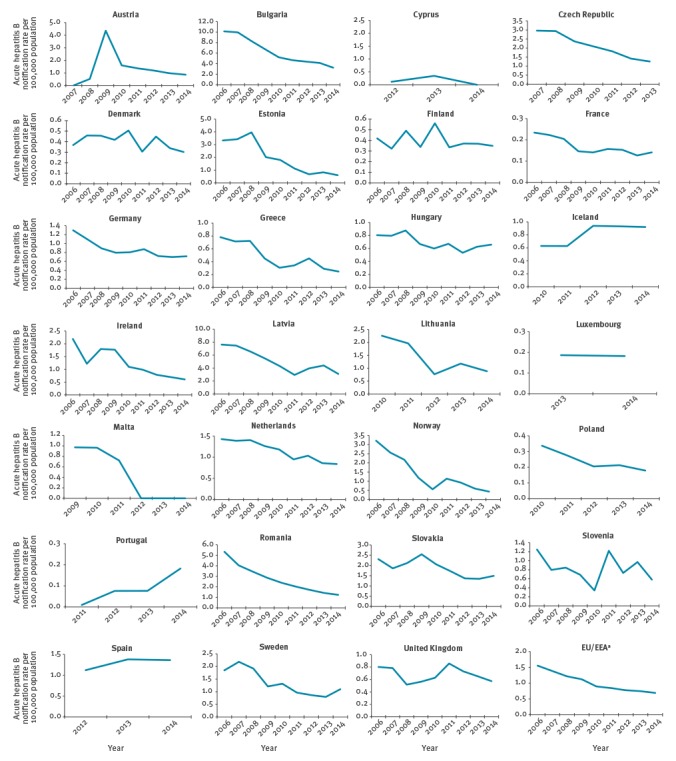
Acute hepatitis B notification rate (per 100,000 population) at country and European Union/European Economic Area^a^ level, 2006–2014 (n = 32,949 notifications)

^a^ EU/EEA include: Austria (2007–2014), Bulgaria (2006–2014), Cyprus (2012–2014), Czech Republic (2007–2013), Denmark (2006–2014), Estonia (2006–2014), Finland (2006–2014), France (2006–2014), Germany (2006–2014), Greece (2006–2014), Hungary (2006–2014), Iceland (2010–2014), Ireland (2006–2014), Latvia (2006–2014), Lithuania (2010–2014), Luxembourg (2013–2014), Malta (2009–2014), the Netherlands (2006–2014), Norway (2006–2014), Poland (2010–2014), Portugal (2011–2014), Romania (2006–2014), Slovakia (2006–2014), Slovenia (2006–2014), Spain (2012–2014), Sweden (2006–2014), United Kingdom (2006–2014).

**Figure 2:**
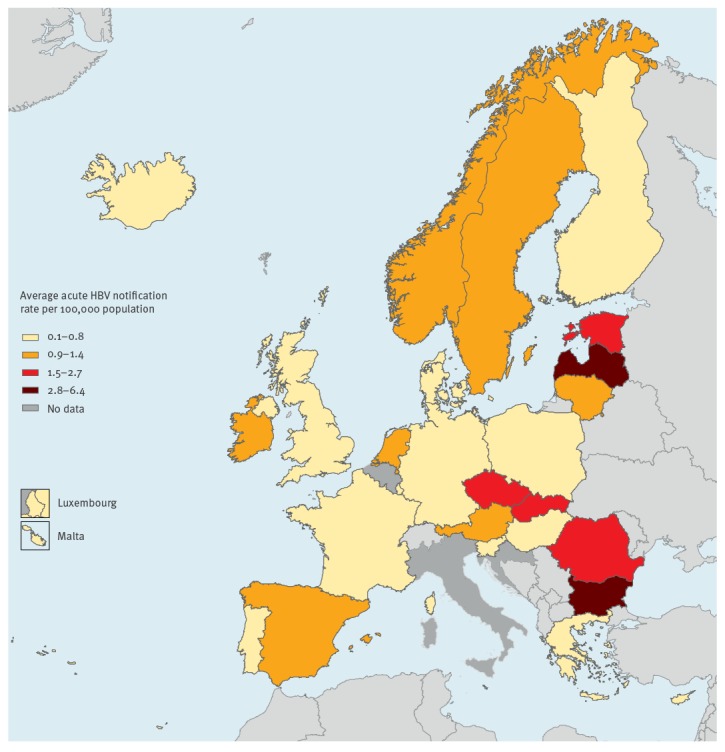
Average acute hepatitis B virus notification rate per 100,000 population in European Union/European Economic Area countries, 2006–2014 (n = 32,949 notifications)

The overall notification rate (EU/EEA level) decreased by 56.2 % between 2006 and 2014, from 1.6 per 100,000 population to 0.7 (Figure 1). In 2014, the rate ranged from 0.0 per 100,000 in Cyprus and Malta to 3.2 per 100,000 in Bulgaria. Not all the countries showed a decreasing trend over the time period; in particular the notification rate increased in Austria (+ 80%), Iceland (+ 50%), Portugal (+ 42.8%) and Spain (+ 27.3%). Figure 2 shows the average acute HBV infection notification rate during 2006–2014 by reporting country. Eastern European countries had the highest values, with the highest rates in Bulgaria (6.3 per 100,000) and Latvia (5.1 per 100,000). At EU/EEA level, the average acute HBV infection notification rate during 2006–2014 was 1.0 per 100,000 population.

HBV: hepatitis B virus.

#### Notification rates for country groups by criterion

**Figure 3:**
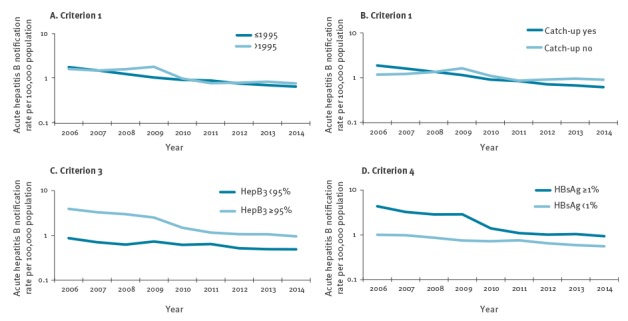
Acute hepatitis B notification rate per 100,000 population (logarithmic scale) by group of countries and criteria, European Union/European Economic Area countries, 2006–2014 (n = 27,288 notifications)

In Figure 3, the group of countries that started a HBV vaccination programme before/in 1995 appear to have a greater reduction**in the acute HBV infection notification rate (-61.1%) compared with the group of countries that started after 1995 (-50%). Similarly, a greater reduction seems to have occurred in the group of countries that implemented a catch-up HBV vaccination programme compared with those countries that had not implemented this strategy (panel B; -68.4% vs -25%). Countries with HepB3 coverage < 95% had a stable trend during 2006–2014 compared with the group of countries with HepB3 coverage ≥ 95%, that seemed to have a more pronounced decrease with -77% (panel C). A similar pattern was observed for criterion 4 (panel D), with a stable trend for the group of countries with HBsAg < 1% and an apparent marked decrease for those countries with HBsAg ≥ 1% (-79%).

HbsAg: hepatitis B surface antigen.

The countries assigned to each of the four grouping criteria described below are listed in Table 1. Cyprus, Luxembourg, Portugal and Spain (n = 4) were excluded from this analysis because they did not report consistently hepatitis B virus infection notification data for at least 5 years, and Denmark, Finland, Iceland, Norway, Sweden and the United Kingdom (n = 6) were excluded because these countries did not have a universal hepatitis B virus vaccination programme between 2006 and 2014. In addition, Hungary was excluded from criteria 3 because this country did not report vaccination coverage data on Centralized Information System for Infectious Diseases (CISID) and Malta was excluded from criteria 4 because HBsAg prevalence data were not available for Malta in the systematic reviews considered in this study [2,19].

**A - Criterion** 1: Countries that started hepatitis B vaccination programme before/in vs after 1995.

**B - Criterion 2:** Countries with/without a catch-up hepatitis B vaccination programme.

**C - Criterion 3:** Countries with three doses hepatitis B vaccine coverage ≥ 95% vs < 95%.

**D - Criterion 4:** Countries with HBsAg prevalence among general population ≥ 1% vs < 1%.

### Statistical assessment of factors and measures potentially affecting HBV infection rates

#### Differences in HBV infection notification rates between groups of countries by criteria

Both at the univariate and multivariate multi-level mixed-effects Poisson regression analyses, no statistically significant differences in the acute HBV infection notification rates were found between the groups of countries for each criterion. For criterion 1 the direction of the association changed from the univariate (IRR: 1.09; 95% CI: 0.35 to 3.37; p = 0.88) to the multivariate analysis. In the latter, countries that started their HBV vaccination programme before 1995 had a lower acute HBV infection notification rate (IRR: 0.61; 95% CI: 0.16 to 2.29; p = 0.46) compared with those countries that started their HBV vaccination programme after 1995. However, results for both analyses were not statistically significant. Concerning criterion 2, countries that implemented a HBV catch-up vaccination strategy did not show a statistically significant higher acute HBV infection notification rate compared with the group of countries that did not implement this strategy, both at the univariate (IRR: 1.30; 95% CI: 0.50 to 3.39; p = 0.59) and multivariate analysis (IRR: 2.37; 95% CI: 0.71 to 7.87; p = 0.16), as well as for criterion 3, where the group of countries with a vaccine coverage ≥ 95% had a no statistically significant higher acute HBV infection notification rate compared with the group of countries with vaccine coverage < 95% (univariate analysis IRR: 1.87; 95% CI: 0.61 to 5.69; p = 0.27; multivariate analysis IRR: 1.87; 95% CI: 0.61 to 5.69; p = 0.27). Finally, countries with a background HBsAg prevalence among general population < 1% showed no statistically significant lower acute HBV infection notification rate compared with the group of countries with HBsAg prevalence  ≥ 1% (univariate analysis IRR: 0.77; 95% CI: 0.28 to 2.13; p = 0.62; multivariate analysis IRR: 0.61; 95% CI: 0.21 to 1.75; p = 0.36).

#### Effect of vaccine coverage on acute HBV notification rate at country and EU/EEA level

Table 2 shows the results of the Poisson regression models assessing the association between HepB3 coverage and acute HBV infection notification rates by country and at EU/EEA level.

**Table 2:** Poisson regression model assessing the association between acute hepatitis B virus infection notification rate and three doses hepatitis B vaccine coverage, at country and European Union/European Economic Area^a^ level, 2006–2014 (n = 22,614 notifications)

Country	Incidence rate ratio (95% CI)	P-value
Austria	0.87 (0.80 to 0.91)	0.01
Bulgaria	0.89 (0.82 to 0.94)	0.01
Czech Republic	0.73 (0.65 to 0.77)	< 0.01
Estonia	0.95 (0.89 to 0.97)	< 0.01
France	0.91 (0.90 to 0.96)	< 0.01
Germany	0.82 (0.80 to 0.84)	< 0.01
Greece	0.86 (0.84 to 0.90)	0.01
Latvia	0.89 (0.87 to 0.94)	< 0.01
Lithuania	0.94 (0.91 to 0.96)	< 0.01
Malta	0.90 (0.85 to 0.96)	0.01
Poland	0.85 (0.77 to 0.96)	0.01
Romania	0.86 (0.84 to 0.87)	0.01
Slovakia	0.92 (0.87 to 0.94)	0.01
Slovenia	0.91 (0.86 to 0.93)	< 0.01
EU/EEA**^a^**	0.90 (0.86 to 0.92)	< 0.01

CI: confidence interval; EU/EEA: European Union/European Economic Area.

Cyprus, Denmark, Finland, Hungary, Iceland, Ireland, Luxembourg, the Netherlands, Norway, Portugal, Spain, Sweden, United Kingdom (n = 13) were excluded in this analysis because these countries did not report both acute HBV cases and HepB3 coverage data for at least five years during the study period.

^a ^EU/EEA countries included: Austria 2007–2014; Bulgaria 2006–2014; Czech Republic 2007–2013; Estonia 2006–2014; France 2006–2014; Germany 2006–2014; Greece 2006–2014; Latvia 2006–2014; Lithuania 2010–2014; Malta 2009–2014; Poland 2010–2014; Romania 2006–2014; Slovakia 2006–2014; Slovenia 2007–2012.

The average EU/EEA HepB3 coverage during 2005–2013 was 91.5%, ranging from 48.6% in France to 99.0% in Czech Republic (data not shown). At the EU/EEA level, HepB3 coverage increased from 90.1% in 2005 to 93.4% in 2013 (+ 3.5%). There were differences at the country level with nine countries having a decreasing trend of HepB3 coverage over the period 2006–2014: Austria (-10.8%), Bulgaria (-1.1%), Estonia (-2.2%), Latvia (-2.8%), Lithuania (-2.3%), Poland (-0.5%), Romania (-2.3%), Slovakia (-2.6%), Slovenia (-1.0%). The overall (EU/EEA) acute HBV IRR decreased by 10% for each 1% increase in vaccine coverage (IRR: 0.90; 95% CI: 0.86 to 0.92; p < 0.01). All countries had a statistically significant association between the decrease of acute HBV IRR and the increase of vaccine coverage. The reduction ranged from a minimum of 5% in Estonia (IRR: 0.95; 95% CI: 0.89 to 0.97; p < 0.01) to a maximum of 27% in Czech Republic (IRR: 0.73; 95% CI: 0.65 to 0.77; p < 0.01), for each 1% increase in vaccine coverage.

#### Trend changes in acute HBV infection notification rates in groups of countries by criteria

The JP regression analysis (Table 3) shows that in the group of countries that started HBV vaccination programme before/in 1995, there was a statistically significant reduction in the acute HBV infection notification rate during the whole study period 2006–2014 (AAPC: -7.4; 95% CI: -11.3 to -3.3; p < 0.01). One JP was also detected in 2010 with the APC that changed from -15.2 (95% CI: -18.8 to -11.4; p < 0.01) in 2006–2010 to -7.4 (95% CI: -11.3 to -3.3; p < 0.01) in the following years. For the group of countries that started HBV vaccination programme after 1995, no JPs were found, and the decrease in the acute HBV notification rate during 2006–2014 (AAPC: -6.8; 95% CI: -9.2 to 1.4; p = 0.09) was not statistically significant.

**Table 3:** Joinpoint regression model output, measuring the change in acute hepatitis B infection notification rate, by group of countries and criteria, European Union/European Economic Area, 2006–2014 (n = 27,288 notifications^a^)

Criterion	Grouping of countries	Joinpoint	Years range	APC (95%CI)	P-value	AAPC 2006–2014 (95%CI)	P-value
1	Started hepatitis B vaccination programme before/in 1995	2010	2006–2010	-15.2 (-18.8 to -11.4)	< 0.01	-7.4 (-11.3 to -3.3)	< 0.01
			2010–2014	-7.4 (-11.3 to -3.3)	< 0.01		
	Started hepatitis B vaccination programme after 1995	No JP	NA	NA	NA	-6.8 (-9.2 to 1.4)	0.09
2	Any kind of catch-up hepatitis B vaccination programme	2011	2006–2011	-8.3 (-17.5 to 1.9)	0.11	-10.4 (-15.3 to -5.2)	< 0.01
			2011–2014	-16.4 (-20.2 to -12.3)	< 0.01		
	No catch-up hepatitis B vaccination programme	No JP	NA	NA	NA	-4.9 (-8.4 to 1.8)	0.19
3	Three doses hepatitis B vaccine coverage ≥ 95%	No JP	NA	NA	NA	-18.1 (-22.0 to -14.0)	< 0.01
	Three doses hepatitis B vaccination coverage < 95%	No JP	NA	NA	NA	-6.3 (-8.8 to -3.6)	< 0.01
4	HBsAg prevalence among general population ≥ 1%	No JP	NA	NA	NA	-19.1 (-23.6 to -14.4)	< 0.01
	HBsAg prevalence among general population < 1%	No JP	NA	NA	NA	-7.1 (-9.2 to -4.9)	< 0.01

APC: annual percentage change; AAPC: average APC; CI: confidence interval; JP: joinpoint; NA: not applicable.

^a^ Denmark, Finland, Iceland, Norway, Sweden and the United Kingdom (n = 6) were excluded from this analysis because these countries did not have a universal hepatitis B virus vaccination programme between 2006 and 2014. In addition, Hungary was excluded from criteria 3 because this country did not report vaccination coverage data on Centralized Information System for Infectious Diseases (CISID) and Malta was excluded from criteria 4 because there was no HBsAg prevalence data for Malta in the systematic reviews [2,19].

The group of countries with any kind of catch-up HBV vaccination programme showed a statistically significant decreasing trend in its acute HBV infection notification rate during 2006–2014 (AAPC: -10.4; 95% CI; -15.3 to -5.2; p < 0.01). One JP was also detected in 2011 with the APC that decreased from -8.3 (95% CI: -17.5 to 1.9; p = 0.11) in 2006–2011 to -16.4 (95% CI: -20.2 to -12.3; p < 0.01) in the following years. On the other hand, no JPs were found for the group of countries without catch-up HBV vaccination programmes, which showed a decreasing trend during 2006–2014 that was not statistically significant (AAPC: -4.9; 95% CI: -8.4 to 1.8; p = 0.19). Concerning HepB3 coverage, no JPs were detected in the groups of countries. Both groups showed a statistically significant decreasing trend in their acute HBV infection notification rate during 2006–2014, with a more marked decrease for those countries with HepB3 coverage ≥ 95% (AAPC: -18.1; 95% CI: -22.0 to -14.0; p < 0.01) than those countries with HepB3 coverage < 95% (AAPC: -6.3; 95% CI: -8.8 to -3.6; p < 0.01). Also for HBsAg prevalence, no JPs were found in the two groups of countries, but both groups showed a statistically significant decreasing trend in the acute HBV infection notification rate during 2006–2014, with a more marked decrease for those countries with HBsAg ≥ 1% (AAPC: -19.1; 95% CI: -23.6 to -14.4; p < 0.01) than to those countries with HBsAg < 1% (AAPC: -7.1; 95% CI: -9.2 to -4.9; p < 0.01).

## Discussion

This study presents surveillance data on the acute HBV infection notification rate at country and EU/EEA level during 2006–2014, analysing the impact of different HBV vaccination strategies, vaccine coverage achieved and the effect of the background HBsAg prevalence. 

All countries had strategies for vaccinating high risk individuals and for prevention-screening (e.g. mother-to-child-transmission), so they were not grouped and compared based on the presence or absence of such strategies [17].

Overall, acute HBV infection notification rates showed a decrease at EU/EEA level between 2006 and 2014, with great variation in rates reported by countries. It is possible that differences in the case definitions used may have contributed to the variation [7] as well as the problem of under-reporting, which has been estimated to be as high as 85% in France [26]. Four countries (Austria, Iceland, Portugal and Spain) had an upward trend over the study period. This finding is consistent with previous reports published in the literature. A study conducted in Spain in 2012 showed an increased HBV incidence between 2000 and 2004 among immigrants [27] and a recent study from Portugal in 2016 indicated a very high HBsAg prevalence among high-risk groups (people who inject drugs (PWIDs) and prisoners) that may have influenced the increase in the HBV reported cases in the country [28]. Both Spain and Portugal have implemented a universal HBV vaccination programme but both implemented these programmes after 1995 and without catch-up strategies, resulting in a proportion of the at risk population remaining unprotected.

A review of HBV surveillance and epidemiology in the EU/EEA showed an increase in the HBV incidence in Austria and Iceland between 1995 and 2005 [29]. While Iceland has not implemented a universal HBV vaccination programme, Austria has a HepB3 coverage < 95%, an HBsAg prevalence among the general population ≥ 1% and has implemented a catch-up HBV vaccination programme.

Even if Norway and Sweden did not implement a universal vaccination strategy, they showed a marked decrease in the acute HBV infection notification rate during 2006–2014 that might be due to the high vaccination coverage reached among PWIDs and their close contacts or related to the decrease in heroin users due to the implementation of a strong-restrictive drug policy [30,31].

Bulgaria, Latvia and Romania reported the highest acute HBV infection notification rates. Eastern European countries have been found to have the highest level of HBsAg prevalence in Europe [2]. Bulgaria, Latvia and Romania have all implemented a HBV universal vaccination programme supported by catch-up strategies, and have attained HBV vaccine coverage ≥ 95%. These countries showed a marked decrease in their notification rates over the study period, suggesting that they have benefited from HBV vaccination programmes, as the greater reduction in rates for countries with a higher rather than a lower HBsAg prevalence shows.

The univariate and multivariate multi-level mixed-effects Poisson regression model indicated no statistically significant differences in the trend of acute HBV infection notification rate between groups of countries assigned to each criterion. One possible explanation for this finding is that the data may have been strongly influenced by singular country profiles. Indeed, Bulgaria, Latvia and Romania, the countries with the highest incidence, belong to the groups that implemented a catch-up strategy, that reached HepB3 coverage ≥ 95% and that had an HBsAg prevalence ≥ 1%. This may have affected the comparison of the trend of acute HBV infection notification rate (during 2006–2014) between groups of countries. In Figure 2, panel C, the group of countries with HepB3 coverage ≥ 95% maintained a higher acute HBV infection notification rate during 2006–2014 compared with the group of countries with a HepB3 coverage < 95%. Overall, the group with HepB3 coverage ≥ 95%, which included countries as Bulgaria, Latvia and Romania, had a marked decrease, but during the whole study period there was no statistically significant difference between the two trends and the multilevel mixed-effects model did not indicate a statistically significant higher acute HBV infection notification rate for the group of countries with a HepB3 coverage ≥ 95% compared with the group of countries with vaccine coverage < 95%.

This limitation, intrinsic in the output of our comparison model, is then nullified by the assessment of the singular acute HBV trends by group of countries assigned to each criterion through the descriptive epidemiology and the JP regression analysis. The latter showed a more marked, and significant, decrease for those countries that started HBV vaccination programme before/in 1995, that implemented a catch-up HBV vaccination strategy and that had HepB3 coverage ≥ 95%, suggesting a positive effect of these factors on the decreasing trend, in particular for those countries with HBsAg prevalence ≥ 1%. Such countries indeed showed a greater reduction in rates than countries with a lower HBsAg prevalence. It should be noted that also these results (e.g. the JPs found) may have been driven by singular country profiles.

The contrasting results of the multi-level mixed-effects Poisson regression model, which finds no difference between groups of countries classified according to HepB3 coverage ≥ 95% vs < 95%, compared to the Poisson regression models, which show significant association between HepB3 coverage and acute HBV infection notification rates, is due to the different outputs of the models and in the inputs given as described in the methods section.

The finding that countries that started their HBV vaccination programme before 1995 experienced a marked decrease in the acute HBV infection notification rate, may be due to the accumulation of vaccinated people over the years and to the long-term protection (herd immunity) provided by the HBV vaccine [8,32]. A study conducted in Italy [11] showed that as a result of the introduction of a universal compulsory HBV vaccination programme in 1991, there was a significant decrease in the HBV infection notification rates in all age groups. In Romania, after the introduction of a routine HBV vaccination programme for children and healthcare workers in 1990 [12], reported hepatitis B incidence declined from 43 cases per 100,000 in 1989 to 8.5 per 100,000 in 2004. A further study conducted in Bulgaria [13], showed that the introduction of HBV vaccine in 1992 resulted in a statistically significant decrease of 82% in the incidence in infants in their first year of life.

It has been reported that HBV catch-up immunisation strategies have also impacted on the HBV epidemiology by increasing herd immunity. A study conducted in Poland [33] observed that after the introduction of the catch-up vaccination programme, the incidence per 100,000 population of newly diagnosed HBV cases in the age group 11–14 years decreased from 1.3 in 2007 to 0.4 in 2011 and had a herd immunity effect among individuals aged 15–24 years. A study conducted in China has shown that catch-up vaccination in children is cost-effective [34]. In the United States, after a catch-up immunisation programme for Alaska Native people, the incidence of acute symptomatic HBV infection in Alaska Natives under 20 years of age fell from 19 per 100,000 in 1981–1982 to 0 per 100,000 in 1993–1994 [35].

Maintaining high HBV vaccination coverage is recognised by WHO as important in national efforts to reduce the burden of HBV infections [8]. The JP regression analysis found that countries with HepB3 coverage ≥ 95% had a greater reduction in the acute HBV infection notification rate compared with countries with a lower coverage. The additional Poisson analysis found a statistically significant correlation between the increase of HBV vaccine coverage and the decrease of acute HBV incidence, showing that as vaccine coverage increases, acute HBV infection notification rates decrease, highlighting the importance of high coverage in achieving the greatest impact.

These findings are consistent with two recent studies in Turkey and Spain. The study conducted in Turkey [36] indicated that the HBV incidence dropped from 12.3 per 100,000 population in 2005, when the HepB3 coverage level was lower than 80%, to 3.6 in 2012, when HepB3 coverage was above 90%. The study in Turkey concluded that both high vaccination coverage and catch-up strategies had a positive impact and suggested that these measures had interacted synergistically. The Spanish study evaluated the association between HepB3 coverage levels and the HBV incidence rate [27], and concluded that the higher the vaccination coverage, the lower the reported incidence of hepatitis B. In groups with vaccination coverage ≥ 70%, the reduction in incidence was twofold higher than in groups with a coverage < 70%.

Limitations of our study are represented by the variation in notification rates at country level; the results of statistical models may have been driven by this variation and by the profile of countries such as Bulgaria, Latvia and Romania (that were in the same groups for all the four analysed criteria), that had a high burden of acute HBV, but also a marked decrease in the notification rates. In addition the heterogeneity of pre-vaccination HBV epidemiology in the EU/EEA may have influenced the impact of the different vaccination strategies analysed and also the results of the statistical models; moreover, because the incidence of acute HBV infection is very low in EU-western countries during the first 15 years of life, the impact of vaccination may will not show for at least 15 years, in contrast to countries with higher HBsAg prevalence where infections may occur earlier in life.

Another limitation is represented by the changes in reporting practices in countries across the period. While many countries were able to use the new EU case definition from 2012 [37], other countries reported cases as defined by their own national case definition or one of the previous EU case definitions; this heterogeneity presents a challenge for the comparison of data between countries. Other key limitations included the incompleteness of data and the under-reporting problem [7]. The incompleteness of the data limited the multi-level mixed-effects Poisson regression analysis, the Poisson regression models assessing the association between HepB3 coverage and acute HBV infection notification and the JP regressions analysis, to 17, 14 and 21 countries, respectively, impairing an understanding of acute HBV epidemiology at EU/EEA level. Although data completeness improved over the reporting period, further work is necessary at regional and national levels to address this issue.

The public health implications for the EU/EEA countries indicated by the findings of our study, are in line with the strategies defined by the WHO for the elimination of HBV as public health problem by 2030 [9]. Among the five core intervention areas to achieve the HBV elimination goal, WHO identified priority actions for countries as the following: the inclusion of HBV vaccine in national childhood immunisation schedules, the implementation of catch-up HBV vaccination strategies and the achievement of 90% vaccine coverage (third dose coverage). Until recently six EU/EEA countries had not yet included HBV vaccine in their national immunisation plan, nineteen did not implement catch-up immunisation strategies and eight countries had a HepB3 coverage < 95%. At the start of 2017, it has been announced that Norway has decided to implement a universal immunisation programme [38].

Vaccination is central to national prevention efforts for HBV, and ending the transmission of HBV in Europe by 2030 will require high vaccine coverage delivered through universal programmes, supported, where appropriate, by catch-up vaccination campaigns, together with a cohesive and coordinated EU strategy. The importance of these interventions is further underlined by evidence of ongoing transmission in Europe and the impact of migration upon HBV epidemiology in Europe [39,40].

In conclusion, our study found a reduction in the acute HBV infection notification rates at EU/EEA level over the period 2006 to 2014, and also found that universal and catch-up HBV vaccination strategies implemented with high coverage are likely to have contributed in this downward trend. Although there are declining trends in the incidence of HBV in many EU/EEA countries, the changing demography, particularly with increasing immigration from countries with intermediate and high HBV endemicity, is relevant as it contributes to 5% of the overall number (EU/EEA level) of HBV cases [40]. Moreover, the evidence of transmission among high risk groups (e.g. PWIDs, men who have sex with men) indicates that there is no room for complacency and this highlights the importance of maintaining prevention and control measures [5].
